# Innovative actions in oceans and human health for Europe

**DOI:** 10.1093/heapro/daab203

**Published:** 2021-12-22

**Authors:** Noortje Pellens, Eline Boelee, Joana M Veiga, Lora E Fleming, Anouk Blauw

**Affiliations:** 1 Faculty of Geosciences, Utrecht University, Princetonlaan 8a, 3584 CB Utrecht, the Netherlands; 2 Deltares, PO Box 177, 2600 MH Delft and Daltonlaan 600, 3584 BK Utrecht, the Netherlands; 3 European Centre for Environment and Human Health, University of Exeter Medical School, Exeter, UK

**Keywords:** innovator, health promotion, oceans, blue space, DPSIR

## Abstract

Innovative actions are local initiatives which leverage the interactions between the ocean and human health to reduce the risks and enhance the benefits for the stakeholders and the natural environment. These initiatives can have strong positive effects on human health and wellbeing as well as on the marine environment. We analysed 150 such innovative actions in Europe. Using a combined case study and survey approach, innovative actions were identified using interviews and content analysis of websites and compiled into a database. Quantitative data were analysed according to the *Drivers, Pressures, State, Impact* and *Response* (DPSIR) framework, guided by selected in-depth interviews. Overall, the innovative actions provided a positive impact on the health of both the ocean and humans through increasing food provision, water quality and tourism opportunities; and addressing environmental issues such as commercial fish stock depletion, pollution and climate change. Innovative actions contributed to meeting various targets of the Sustainable Development Goals (SDGs) 3, 13 and 14. These actions played a potential role ahead of and alongside policy. Some of the innovative actions may have potential to be put in place elsewhere. Such up-scaling would need to be adapted to local circumstances and could be facilitated by an innovative action exchange platform.

## INTRODUCTION

The coasts, seas, global oceans and humans are connected in a variety of ways (Bowen *et al.*, 2014). Oceans provide vital ecosystem services that are crucial for human health and wellbeing, but also pose threats. The positive and negative interconnections between human health and ocean activities are complex (Fleming *et al.*, 2021). People can not only impact the ‘health’ of the ocean through pollution and overexploitation but also play a key role in conservation efforts. In turn, healthy oceans bring more benefits for human health and wellbeing (Fleming *et al.*, 2019). These insights are largely based on research in the United States, whereas far less is known about oceans and health interactions in Europe, which has its own characteristic set of challenges and opportunities (Fleming *et al.*, 2014). This article is a first attempt to unlock this by providing an entry point to a wealth of Europe-based initiatives related to ocean and human health interaction.

The importance of oceans to human health is recognized and reflected in the United Nations Sustainable Development Goals (SDGs), specifically the SDG 14—*Life below Water*. In Europe, the most important ocean benefits to human health are related to livelihoods, food provision, pharmaceuticals, and recreational spaces (European Marine Board, 2013). These contribute to human physical health as well as to mental wellbeing.

Food provision includes fisheries, aquaculture and seaweed cultivation. Seafood is an important source of protein, vitamins and minerals; compared to other types of food, it is very rich in concentrations of omega-3 fatty acids which can prevent chronic diseases (European Marine Board, 2013; Moore *et al*., 2013). However, overfishing is a great threat to the European marine environment (European Marine Board, 2013). European fish stocks have greatly declined because of unsustainable fisheries. In 2018, in the Mediterranean and Black sea, an estimated 88% of the assessed stocks were overfished (European Environmental Agency, 2018).

In addition to overfishing, important environmental issues related to oceans and human health include climate change and pollution. The ocean plays a key role in climate regulation, producing oxygen and absorbing carbon dioxide, although currently, it is reaching the buffering limit, leading to ocean acidification. The most visible effect of climate change is the increase in extreme weather events (European Marine Board, 2013), while rising water temperatures lead to changes in fish migration and may create more frequent harmful algal blooms (Fleming *et al.*, 2015). Solid waste (including plastics), chemical substances and pathogens may pollute and contaminate coastal waters and the marine food chain (Landrigan *et al.*, 2020).

The search for pharmaceuticals and other natural products in the marine environment has become increasingly important for finding treatments for diseases such as cancer and AIDS, and for solving challenges related to antibiotic resistance (European Marine Board, 2013; Fleming *et al*., 2015). This is highly dependent on a healthy, biodiverse marine ecosystem.

Coastal and maritime tourism is a key area for the European Blue Growth Strategy, with high potential for sustainable wealth and job creation. For example, the Mediterranean is an important global destination, attracting one-third of the international tourists worldwide (UNWTO, 2015). Coastal and maritime areas are key tourism attractions; and ecosystem assets such as clean beaches and good coastal water quality are essential (Börger *et al.*, 2021). In turn, tourism may also impact the health of maritime ecosystems, for instance by generating additional pressures from the increased production of solid waste and wastewater, which can lead to marine pollution and further degradation of coastal areas (Börger *et al*., 2021).

The health and wellbeing benefits of spending time in coastal and marine environments (a.k.a. *blue spaces*) include improved self-esteem, social confidence and resilience, as well as a sense of environmental connectedness (Britton *et al*., 2020). People perceive marine and other aquatic environments to be among the most restorative, and they are more likely to engage in physical activities there (Korpela *et al*., 2010; Wheeler *et al*., 2015; White *et al*., 2020). Mental health benefits, such as feelings of calmness and revitalization, are found to be stronger in coastal environments than in urban green spaces or the countryside (Britton *et al*., 2020). These factors could contribute to the prevention and treatment of non-communicable chronic diseases (e.g. diabetes, cardiovascular disease), which make up an estimated 80% of healthcare costs in Europe (European Commission, 2019).

To manage human health and environmental issues in the ocean, integrated policies are needed. However, the two domains of the marine environment and human health are largely disconnected, both in science and policy (McMeel *et al.*, 2019). Moreover, the significant impacts that humans have on the health of the ocean result from drivers and pressures often originating from non-maritime activities. It is crucial that these interactions are better understood and managed through local, regional, and global political action and stakeholder participation (Fleming *et al*., 2019). Thus, responses that address the ocean and health interactions beyond these two separate domains are necessary.

Convincing case studies are needed to improve understanding and illustrate the links between oceans and human health. In turn, these can serve as inspiring examples and guide future integrated actions and development of policies at local, regional and European levels (Fleming *et al*., 2014). This article hopes to strengthen the recognition of the interdependency of the oceans and human health, and foster, as well as connect, future initiatives by presenting ‘innovative actions’ in Europe, assessing how these directly or indirectly promote the benefits of the ocean to human health and wellbeing, in a sustainable way. This adds to the body of knowledge on the benefits of high-quality blue spaces in relation to human health (Maller *et al*., 2006; Ballesteros-Olza *et al.*, 2020; White *et al*., 2020).

An innovative action is defined here as a local initiative (started by ‘innovators’) that makes use of the interactions between the ocean and human health to both reduce risks and enhance benefits for both stakeholders and marine ecosystems. Excluding practices that are institutionalized or formalized on a large scale, these actions address the public issues of oceans and human health from the bottom up, that is, outside most policies and regulations.

## METHODOLOGY

### Theoretical framework

Innovative actions in Oceans and Human Health can be considered as *Responses* in the DPSIR (Drivers, Pressures, State, Impact, Responses) theoretical framework (Yee *et al.*, 2012; Lewison *et al.*, 2016; Boelee *et al.*, 2019). These *Responses* may change *Drivers*, reduce *Pressures*, act to preserve the environmental or human *State*, or minimize negative and increase positive *Impacts* (Supplementary Appendix S1).

Describing the impact of innovative actions on environmental issues and ecosystem services provides insight into how they improve environmental and social conditions, respectively (Ford *et al.*, 2015). However, additional analysis is required to translate that impact into concrete environmental and human health benefits. Thus, the innovative actions were assessed in terms of their contributions to the 17 Sustainable Development Goals (SDGs) (United Nations, 2021). The SDGs have high societal relevance as guiding principles in the transition towards a sustainable global future. Specific targets in three SDGs relate directly to the oceans and human health, notably in SDG 3—Good Health and Well-being, SDG 13—Climate Action and SDG 14—Life below Water. To fully deliver these, or rather, all SDGs, the health of both humans and the environment must be achieved (Eckermann, 2016; Spencer *et al*., 2019). Ideally, the emphasis must be on the prevention of human disease and environmental destruction rather than on subsequent treatment and remediation (van den Bosch and Bird, 2018).

The European Union is progressing well on almost all SDGs (Eurostat, 2018), though from 2020 this is impacted by the COVID-19 pandemic (Eurostat, 2021). Interestingly, while non-communicable chronic diseases make up for 80% of the healthcare costs, only 3% of health budgets is spent on prevention (European Commission, 2019). Member states score lowest on their progress for the SDG 14—the marine environment (European Commission, 2019). Ideally innovative actions in oceans and human health will address the impacts of climate change as well as other environmental threats, and at the same time promote healthy behaviour and prevent non-communicable diseases (WHO, 2017).

### Approach

Quantitative and qualitative methods were used, applying a combined case study and survey design. Our purposive sampling method limited the sample to innovative actions in Oceans and Human Health in Europe according to pre-determined criteria (Kothari, 2004). Innovative actions were mainly collected on the internet, using various search engines (Google, Ecosia, DuckDuckGo) to find relevant websites, social media channels (Facebook, Twitter, and LinkedIn) and online newspaper articles in English, Dutch, Spanish and Portuguese. Search terms included: ocean(s), health, mental health, beach, blue spaces, green spaces, benefits, ecosystem services, initiative, innovation and citizen science. Colleague researchers and their networks helped to identify additional cases.

The innovative actions were registered on an online form (Supplementary Appendix S2) by the researchers, and, in a few cases, by the innovators themselves. Data collected included: location, environmental issues addressed and ecosystem services (including health and wellbeing) provided. During the data collection process, the inventory of actions was monitored to ensure that the broadest possible range of topics was covered. Whenever a gap was identified in either topic or geographical area, additional effort was made to search for complementary innovative actions, that is, purposive sampling (Kothari, 2004).

Additional in-depth information was gathered through sixteen semi-structured interviews with innovators of a wide range of actions as well as experts in research and policy on oceans and human health. These semi-structured interviews complemented the quantitative information in the databases and provided more insight into the impacts of innovations, in line with the concept of an exploratory study, and to identify knowledge gaps and topics for further research. Pre-tested questionnaires were used (Supplementary Appendix S3), in accordance with the guidelines for ethical conduct of Utrecht University. The innovators provided more details on their innovative actions with limited information available online, and were asked questions about their motivations, likely success factors, perceived impact and options for upscaling, as well as suggestions for additional actions. The interviews with experts helped to gain insight into the role of innovative actions in policy making and their potential impact.

### Data analysis

The innovative actions were assessed quantitatively by ‘content analysis’ of their web pages and additional (social) media such as blogs and online videos (Krippendorff, 2004; Kim and Kuljis, 2010). The information entered in the online data form (using Formdesk) was imported into both Excel and SPSS (Krippendorff, 2004); and further categorized and cross-tabulated. Each innovative action was classified as a *Response* to either *Drivers*, *Pressures*, *State*, or *Impacts* according to the concepts in Supplementary Appendix S1. The resulting database provides an interesting overview of innovative actions across Europe (Supplementary Appendix S4). Content analysis revealed the role of innovative actions within the Oceans and Human Health interactions, which environmental issues they covered, which health benefits they generated, the Sustainable Development Goals they addressed, and where they were located.

As much as possible, content analysis was used to analyse the data into quantitative categories for comparison. Spider diagrams helped to visualize the application of the DPSIR framework to environmental issues and ecosystem services in an integrated manner.

Subsequently, qualitative data from the interviews were recorded, transcribed, coded and analysed using the NVivo programme version 12.6 (Hennink *et al.*, 2011). Inductive and deductive coding were applied by formulating codes based both on the topics of the interview guide and on issues raised by participants.

## RESULTS

A total of 150 innovative actions were collected, categorized and analysed, in addition to 16 in-depth interviews that were held during a period of three months. The collected innovative actions were very diverse (presented in full in Supplementary Appendix S4 and grouped in Table 1). The listed actions proposed to contribute to increased scientific knowledge and research on ocean health, pollution and biodiversity, or to reduce pressure on commercial fish stocks. Some of them addressed pollution directly, such as voluntary clean-up initiatives. Others facilitated access to coastal areas with inclusive recreational activities that strengthened the physical health and mental wellbeing of residents and visitors. Other actions created protected areas, spread awareness of climate change and environmentally friendly behaviour and optimized technologies for biodiversity-friendly fisheries, or contributed to knowledge of the ecological status of marine and coastal areas via citizen science data collection.

**Table 1:** Types of innovative actions in oceans and health in Europe

Type of actions	Description
Early warning systems	Attempt to forecast, for example, targeted at bathing water quality, to reduce the risk of ingesting contaminated water or exposure to harmful substances during bathing activities.
Citizen-science	Involve citizens in research projects to monitor issues such as coverage of seagrasses, coral bleaching, water quality, microplastics and biodiversity.
Sustainable food provision	Explore sustainable fishing practices that reduce pressures on over-exploited species and focus on alternative species for consumption; also, the development of alternative food sources from the ocean such as seaweed cultivation.
Conservation and restoration	Address the state of the environment by enhancing biodiversity, restoring coral reefs, mangroves, and other marine habitats, setting protected areas and applying new technologies such as artificial reefs. Often these projects have recreational or citizen science components to them, where volunteers can help with replanting or monitoring in the field.
Ecotourism	Offer an alternative to mainstream or mass tourism in coastal areas. An example is a whale-watching certificate for tourist operators that protects the animals as well as promotes human safety. Other ecotourism activities are sustainable sailing expeditions and coastal wellness retreats, or a combination with citizen-science such as tracking wildlife observations during blue tourism excursions.
Environmental education	Promote environment-friendly behaviour and enhance ocean literacy. These include environmental classes in schools, interactive outdoor activities and awareness campaigns. Examples are education programs, film competitions and art programs.
Plastic waste clean-up and recycling	Prevent waste disposal in the marine environment as well as the removal thereof and the re-use of plastic waste as a resource for new products with recreational value. Examples are clean-up activities, 3D printed coastal recreational spaces and benches, surfboards, clothes or eco-bricks for sustainable housing.
Marine Biotechnology	Investigate the potential of biomedical compounds in the marine environment that can be used in the treatment of diseases (e.g. cancer, diabetes or Alzheimer) or provide novel drugs in the face of increasing antimicrobial resistance.
Mental health	Use the restorative effect of the ocean in interactions with the ocean for people suffering from mental health issues like depression, anxiety, stress or PTSD. Activities include sailing, surfing, and meditation at sea for improving mental wellbeing. Adaptive programs are also created to include people with physical or mental disabilities.
Adaptive rescue services	Provide medical training for tourism operators and first responders in rescuing surfers, and post-flood rescue resources.
Renewable energy production	Enable renewable energy production from waves or tides. These are local solutions that can drastically reduce the dependency on fossil fuels, reduce CO_2_ emissions and are sometimes used in combination with spaces for recreation and enhancing biodiversity.

**Fig. 1:**
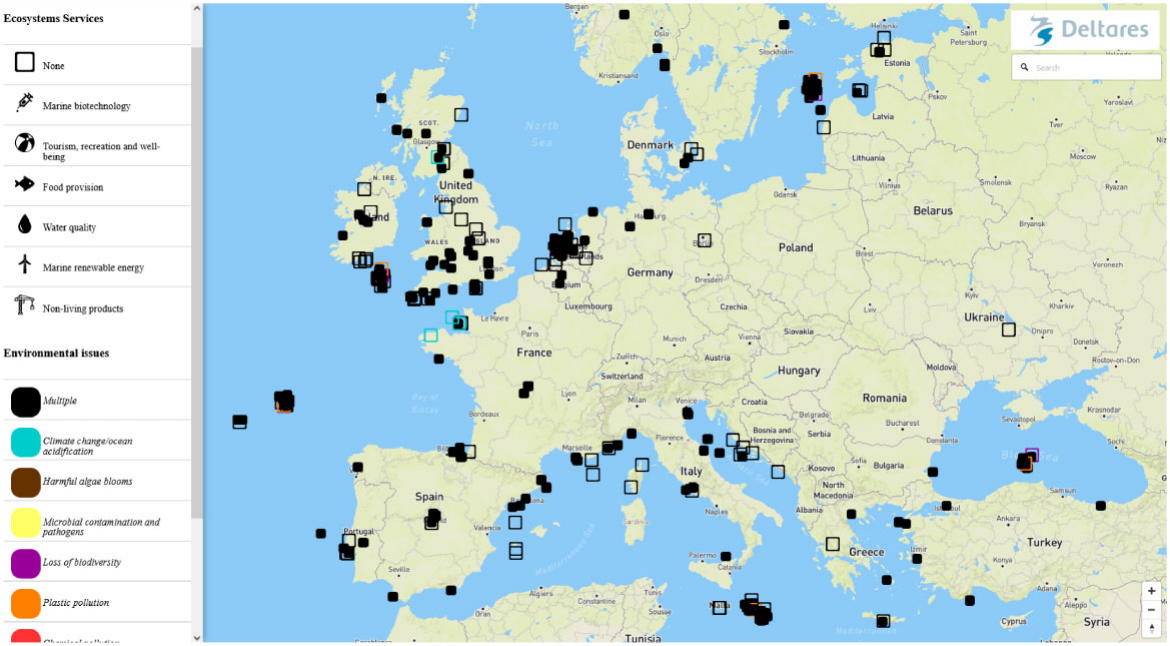
Screenshot of interactive map of innovative actions in oceans and human health in Europe.

The geographical distribution of the innovative actions across the European sea basins was unequal (Figure 1). Most cases were found in the North-East Atlantic, North Sea and Mediterranean Sea, whereas least cases were identified in the Black Sea. Possible explanations for this are the bias in language and level of internet use.

### Environmental impact

Each innovative action addressed one or more specific environmental issues. The actions mostly targeted ‘loss of biodiversity’ (33%), ‘plastic pollution’ (31%), ‘commercial fish stock depletion’ (22%) and ‘climate change/ocean acidification’ (19%). This was followed by ‘chemical pollution’ and ‘microbial contamination/pathogens’ (13% each), whereas ‘harmful algal blooms’ (9%) and ‘eutrophication’ (7%) were the least frequently addressed.

The interviews with innovators provided illustrations of how their actions had positive impacts on the environmental quality of coasts and waters through clean-up actions.

The nets collected by Healthy Seas are not sent into landfills or burned. Instead, they are recycled to create high-quality consumer products, like carpets, socks, and swimwear. In the first 6 years of operation, more than 453 tons of ghost nets have been collected and sent for recycling, the equivalent of 3 blue whales. (spokesperson for Healthy Seas, January 2019).

A key component in the area of environmental impact was achieved by education, raising awareness and creating so-called ‘ocean literacy’, that is, the understanding of the influence of the ocean on humans and our influence on the seas and ocean (Uyarra and Borja, 2016). Environment-friendly or pro-environmental behaviour was promoted by connecting people to the marine environment and enhancing their understanding of its value.

Having kids realize that adventure is right on their doorstep and closer than they think, makes them see nature as a playground and lean more towards the environment. (spokesperson for SUP-Kids, January 2019).

Positive environmental co-benefits were also reported through the establishment of protected areas by innovative actions. One example is Vies Braves, which designed a network of open water swimming itineraries along the coast of Catalonia, consequently restricting these spaces from mass tourism and navigational traffic.

We protect the sea during the high season by our coverage of protected itineraries, preventing anchoring ships from damaging the seafloor. Moreover, we bring people closer to the sea through intimate contact with nature, promoting healthy swimming and sports activities in a safe and conscious way. (spokesperson for Vies Braves, February 2019).

### Impacts on human health

The health impact of innovative actions can be determined via the ecosystem services that they offer (multiple answers possible). Ecosystem services are the benefits people obtain from ecosystems (MA, 2005). The most common ecosystem services that the innovative actions helped to provide were in the combined category of ‘tourism, recreation and wellbeing’, targeted by almost three quarters of the actions (72%). Around a third of the actions addressed ‘water quality’ (34%) and ‘food provision’ (29%), whereas only a few covered ‘biotechnology’ (6%) or ‘marine renewable energy’ (3%).

The innovators described their impact on human health in a variety of ways. They contributed to spreading awareness and knowledge about health and safety in coastal and marine areas within an ocean literacy framework (Uyarra and Borja, 2016). Their actions reduced the health risks associated with poor water quality or accidents in water activities. Swim Guide was an initiative committed to collecting data and spreading knowledge on water quality.

People love to swim, and they love their water bodies. They want to know what the water quality is and whether they can swim there and then. Basically, everyone that calls us asks these same questions, so we want to help them with this information. (spokesperson for Swim guide, February 2019).

Aside from physical gains, innovative actions provided a wide range of mental health and wellbeing benefits, both through health promotion and treatment, at times through the lens of ‘social prescribing’ (Husk *et al*., 2020). These actions made use of the calming and relaxing effect of the ocean, and particularly focussed on vulnerable people such as victims of war, people with traumas and mental or physical disorders, people with autism, or people struggling with an addiction. Mental health was then reportedly improved by reduction of depression and anxiety, boosting self-confidence, and improving self-esteem, from interacting with the marine environment, though it is hard to quantify these effects (White *et al*., 2020).

Children that are unhappy are often isolated. Depression and anxiety are made worse or caused by isolation. Breaking this isolation is a big part of the therapy. (spokesperson for The Wave Project, January 2019).

### Contribution to sustainable development goals

**Fig. 2:**
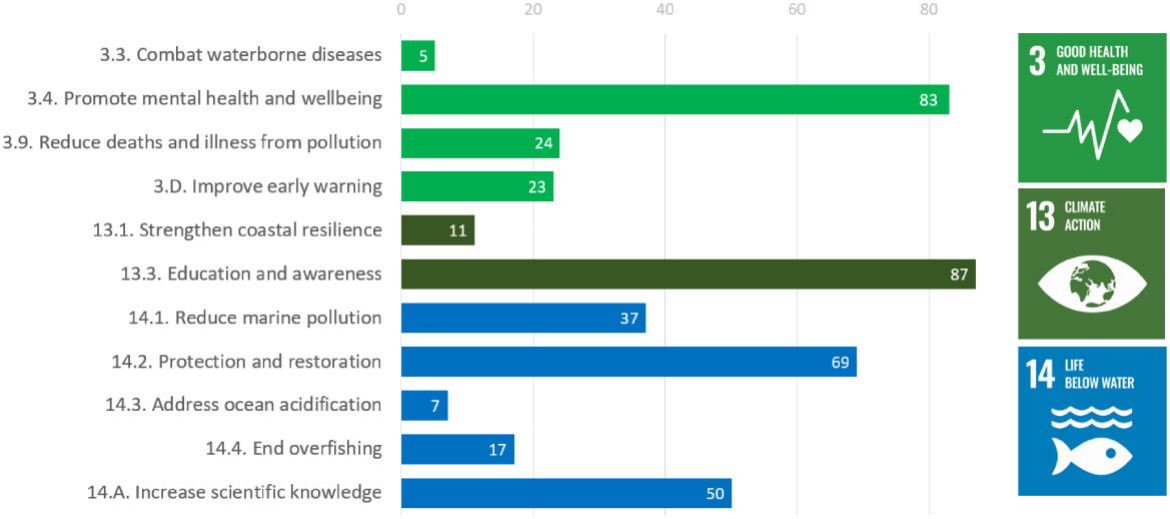
Contributions of 150 innovative actions on oceans and human health in Europe to SDG targets.

The innovative actions contributed to one or more sustainable development goals (SDG) targets that are important focus points of the European Union (EU), addressing important health and environmental issues (multiple answers possible, Figure 2). Innovative actions in environmental education, but also those in conservation and restoration, clean-up or citizen science (types of actions in Table 1) contributed in particular to SDG target 13.3 ‘education and awareness for climate action’ (87 out of 150 innovative actions). Actions such as sports and therapy at sea (type ‘mental health’), rescue services and ecotourism contributed to target 3.4, ‘promotion of mental health and wellbeing’ (83 out of 150) within a social prescribing and health promotion framework. Conservation efforts including restoration of coastal and marine habitats, and clean-up activities directly contributed to SDG target 14.2 ‘protection and restoration of marine ecosystems’ (69 of 150 actions), whereas for instance citizen science helped towards ‘increasing scientific knowledge’ (target 14.A; 50 actions).

### Role of innovative actions as responses

In Supplementary Appendix S4, all identified innovative actions are grouped according to the type of *Response.* More than half of the innovative actions (78 out of 150) were classified as *Responses* to *State*. These are split into those addressing the environmental *State* (e.g. water quality or biodiversity) and those addressing the human *State* (e.g. affected by a chronic disease). Parallel to this, the 29 *Responses* to *Pressures* (20% of the actions) were also distinguished between those addressing environmental *Pressures* (5) and those aimed at human *Pressures* (24). Environmental *Pressures* are linked to emissions, pollution and extractive disturbances, while human *Pressures* are determined by lifestyle factors, for example, in consumption, transportation or housing. The group of 29 *Responses* to *Impact* encompassed actions targeting ecosystem services such as safe seafood, recreational spaces, and climate regulation. Actions addressing *Drivers* (14) were under-represented as *Drivers* are usually targeted by policy and regulations, and much less through local action.

Within the categories of the DPSIR framework, innovative actions addressed various environmental issues; this is discussed in Supplementary Appendix S5.

### Potential for upscaling

The interviews with innovators provided insightful perceptions of the role that innovative actions can play in policy making, and whether they could be upscaled. In general, according to most innovators, the impact of the innovative action was limited to the local scale at which they were implemented. They suggested that international upscaling could increase the overall impact on the marine environment and human health. Although upscaling requires more resources, it can be efficient to scale up and expand to places with similar issues, inspiring people and preventing the duplication of efforts in other places. The innovators were interested in learning from others and sharing experiences.

You could potentially have bigger impact by working out which organizations are doing similar things in different areas and collaborate in that way. You'll be able to scale up much faster and create much more effective impact. (spokesperson for Exxpedition, January 2019)

One way of upscaling is to set up overarching collaborations and partnerships that share knowledge and experiences, while making use of the local resources available. Such a collaborative and network approach is yet to be developed, but it could strengthen existing innovative actions by connecting them with similar initiatives and support cross-country learning. Nonetheless, some innovators stressed that if the actions are to be scaled up, they would need to be identified and supported locally, and adapted to the local circumstances. Innovators have in-depth knowledge of the regional socio-economic, cultural, environmental and geographical context that could help in the coordination and upscaling efforts.

## DISCUSSION

In this inventory, innovative actions targeting ‘loss of biodiversity’, ‘plastic pollution’, ‘climate change’ and ‘commercial fish stock depletion’ were the most abundant. A possible explanation for why the actions covered some environmental issues and ecosystem services more than others, is that certain issues appeal more to people to act on than others (Davison *et al.*, 2021). As assessed by Hartley *et al*. (2018), plastic pollution is widely visible to European visitors to the coast and raises high concern. Therefore, issues such as ‘marine litter’ and ‘loss of biodiversity’ are rather easy to grasp and incite emotions, whereas for instance ‘harmful algal blooms’ and ‘microbial contamination’ may be less so.

Furthermore, some issues, for instance ‘eutrophication’ and ‘harmful algal blooms’, are more challenging to address through concrete actions by innovators. Additional quantitative research is required to understand the (gaps in) thematic coverage of the actions (Depledge *et al.*, 2013). Nevertheless, those issues that tend to trigger more attention can be further exploited given their potential to have over-spilling effects for other issues and to promote overall healthier oceans. For example, tackling marine litter, including lost and discarded fishing gear, can prevent ghost fishing that affects biodiversity.

Health and wellbeing benefits were generated through the provision of ecosystem services such as ‘tourism, recreation and wellbeing’, ‘food provision’ and ‘water quality’. Although the positive impact of oceans and coasts on human health is difficult to quantify, our findings are in line with the positive relationship between proximity to beaches and general health reported in Barcelona and elsewhere (Ballesteros-Olza *et al*., 2020; White *et al*., 2020). This effect may be reinforced through interventions aimed at increasing biodiversity, or restoration efforts, as proximity to and interaction with nature are beneficial to human health, including possibly prevention and treatment of chronic disease (Maller *et al.*, 2006; White *et al.*, 2020).

The SDG targets that these innovative actions most contributed to were ‘education and awareness’ (SDG target 13.3), ‘mental health and wellbeing’ (SDG target 3.4), ‘protection and restoration’ (SDG target 14.2) and ‘increased scientific knowledge’ (SDG target 14.A). These contributions came mainly from actions on environmental education, citizen science, conservation and restoration, clean-up activities, sports and therapy, rescue services, ecotourism and social prescribing (Table 1). These innovative actions covered some of the targets that are currently addressed least successfully in Europe by traditional top-down approaches (European Commission, 2019). This indicates a large potential impact of the innovative actions towards achieving SDG targets, by sustainably promoting physical and mental health, increasing awareness and resilience of climate change, and reducing marine issues such as pollution and overfishing. The innovative actions seem to illustrate an especially strong potential benefit for mental health. This had been highlighted before by various authors (Nichols, 2014; Britton *et al.*, 2020) and merits more attention in science, policy and practice.

The innovative actions can be considered as *Responses* in a DPSIR framework, thus addressing the gap identified by Lewison and colleagues, who argued that ‘*a key area for development focuses on the R in DPSIR*’, beyond policy responses (Lewison *et al.*, 2016). The actions can inspire and play a potential role ahead of and alongside policy. As mentioned in the Introduction, the two policy domains of oceans and health are often disconnected (McMeel *et al.*, 2019). The innovative actions in this inventory have the potential to bridge this divide. They addressed local issues and helped to create awareness of the oceans and human health linkages by storytelling and demonstrating success. The actions in this inventory demonstrated many linkages between oceans and human health, provided examples of what really works, and thus paved the way for more in-depth case studies.

The actions helped to spread awareness by way of storytelling. They were hopeful tales that engaged stakeholders, contributed to increased understanding and could shift the focus away from health threats to the benefits from interacting sustainably with the ocean. Hence, many innovative actions could also complement policymaking, in an effective bottom-up approach. Moreover, innovative actions can test approaches ahead of policy and on a small scale.

A first step towards upscaling could be a systematic exchange of practices, for instance on a platform where innovators can meet, develop networks and share both knowledge and expertise. Such a platform could also serve as a compelling way of engaging people, helping them to understand the many linkages between oceans and human health. It could also inform policy makers and local governments of the potential of innovative actions to increase the positive health impacts of oceans. The typology of actions in Table 1 can be used as a guide to identify and prioritize the most suitable local options.

## CONCLUSIONS AND RECOMMENDATIONS

The rich diversity in innovative actions demonstrated how successful initiatives matched local context, had access to local resources, and, in many cases, took a collaborative and network approach. We recommend the establishment of an exchange platform to support other innovators, as well as scientists and policy makers, in initiating and upscaling innovative actions and sharing knowledge and good practices. Such a platform would fit well in the EU Bioeconomy Strategy European Union (2018), which aims to unlock the potential of seas and oceans, among others by research and innovation. Furthermore, it would align well with the European Green Deal European Commission (2021) striving for a sustainable blue economy by strengthening conservation efforts, coastal resilience and enhanced interactions—to benefit human wellbeing.

More scientific research is recommended to measure the effectiveness of innovative actions, the link with behaviour change and social prescribing (Husk *et al*., 2020), and to identify key factors for success. Increased understanding of beneficial interactions between ocean health and human health can lay the foundation for more integrated policies and decision making; this in turn can lead to better coastal management and marine conservation efforts with the promotion of better health and wellbeing for European citizens.

This first inventory has shown that the many innovative actions have enormous potential to, directly and indirectly, promote the benefits of healthy oceans to human health and wellbeing. Further research is needed around the potential beneficial sustained impact on mental health and wellbeing of such interactions. Innovative actions can improve the state of the environment by raising awareness of environmental issues and the importance of ocean health. They can play a big role in health promotion through providing ecosystem services that translate into health and wellbeing benefits, and they can contribute to achieving key SDG targets in Europe.

## Supplementary Material

daab203_Supplementary_Data
