# Supplementary material for: Innovative actions in oceans and human health for Europe
Source: Health Promot Int. 2021 Dec 22;38(4):daab203. doi: 10.1093/heapro/daab203 (PMC10405041; doi:10.1093/heapro/daab203)
Supplement: daab203_Supplementary_Data [file daab203_supplementary_data.zip › InnovativeActionsOceansHealth_Appendix1new.docx]

**Title**

Innovative actions in Oceans and Human Health for Europe

# SUPPLEMENTARY APPENDIX 1

Key concepts for classification of innovative actions in oceans and human health.

The innovative actions have been classified into categories of the *Drivers, Pressures, State, Impact* and *Responses* framework.

| DPSIR category | Key concepts |
| --- | --- |
| ***Drivers*** |  |
| · Socio-economic | Coastal construction, tourism, agriculture, industrial activity, fishing industry, transportation, fossil resource extraction, population growth, governance, equity |
| · Natural | Natural ecosystem fluctuations, climate change, weather conditions |
| ***Pressures*** |  |
| · Environmental | Atmospheric emissions, microbial and chemical pollution, solid waste, extractive disturbances |
| · Human | Consumption patterns, choice of housing, conservation behaviour |
| ***State*** |  |
| · Environmental | Marine ecosystem: pollutants, biodiversity, ecosystem health, water quality |
| · Human | Human physical and mental diseases and health, demographics |
| ***Impact*** |  |
| · Ecosystem services | Clean water, provision of seafood, opportunities for recreation and tourism, climate regulation |
| · Human health benefits | Physical and mental well-being, life expectancy, economic benefits, social and cultural benefits |
| ***Responses*** |  |
| · Driver level | Marine industry policies, transportation and emission policies |
| · Pressure level | Resource management, coastal planning, waste disposal regulations |
| · State level | Restoration, conservation, monitoring |
| · Impact level | Mitigation, compensation, education and awareness |
