# Supplementary material for: Innovative actions in oceans and human health for Europe
Source: Health Promot Int. 2021 Dec 22;38(4):daab203. doi: 10.1093/heapro/daab203 (PMC10405041; doi:10.1093/heapro/daab203)
Supplement: daab203_Supplementary_Data [file daab203_supplementary_data.zip › InnovativeActionsOceansHealth_Appendix2new.docx]

**Title**

Innovative actions in Oceans and Human Health for Europe

# SUPPLEMENTARY APPENDIX 2

SOPHIE Online Form

| 1. | **Case-study title** |  |
| --- | --- | --- |
| 2. | **Case-study location** E.g. city, country, region where the case-study was implemented |  |
| 3. | **When was the case-study performed?** Please provide year(s) or starting date of the initiative |  |
| 4. | **Scale of implementation** | local |
|  |  | national |
|  |  | sea basin |
|  |  | Europe |
|  |  | global |
| 5. | **Regional Sea basin** Please select the relevant basin where the case-study was implemented. If outside of Europe please select "other" | Baltic Sea |
|  |  | North Sea |
|  |  | Celtic Sea |
|  |  | North East Atlantic |
|  |  | Mediterranean Sea |
|  |  | Black Sea |
|  |  | other |
| 6. | **Environmental issues** What are the issues directly addressed by the case-study? | Climate change, ocean acidification |
|  |  | Commercial fish stocks depletion |
|  |  | Loss of biodiversity |
|  |  | Eutrophication |
|  |  | Harmful algae blooms |
|  |  | Plastic pollution |
|  |  | Chemical pollution (e.g. oils spills, pharmaceuticals, pesticides, heavy metals) |
|  |  | Microbiological contamination/pathogens |
|  |  | None |
|  |  | Other: |
| 7. | **Ecosystem services** Which marine services and uses can be improved by the case-study? | Food provision (e.g. fish, shellfish) |
|  |  | Marine biotechnology (e.g. medical compounds, cosmetics) |
|  |  | Water quality (e.g. for bathing, aquaculture, desalination) |
|  |  | Tourism, recreation and well-being |
|  |  | Marine renewable energies |
|  |  | Non-living products (e.g. minerals, oil, gas) |
|  |  | None |
|  |  | Other: |
| 8. | **Case-study description** Please provide a short description of the solution, including main objectives, approach and key outcomes |  |
| 9. | **Type of initiative** Please select the main type of approach underlying the case-study | Awareness campaign/Education |
|  |  | Practice/Action |
|  |  | Study/Research |
|  |  | Monitoring/Information tool |
|  |  | Multidisciplinary collaboration |
|  |  | Policy/Regulation |
|  |  | Voluntary agreement |
|  |  | Other: |
| 10. | **Lessons learnt** What would you advise to people that would like to try a similar action in their area? When is this type of solution particularly useful and when not? |  |
| 11. | **Similar applications** Do you know other applications of this type of solution? |  |
| 12. | **Keywords** Please provide 3 - 5 keywords that characterize this case-study |  |
| 13. | **Is there more information available on internet?** Please provide website address |  |
| 14. | **Who are the main promotors of the initiative?** Please indicate the entity(ies) responsible for the implementation of the case-study |  |
| 15. | **Who can we contact for further information?** Please provide contact person and/or e-mail address |  |
| 16. | **Is there documentation available on this case study?** Please upload relevant documents |  |
| 17. | **May we contact you for further information?** (if so, please provide your name and e-mail address) |  |
| 18. | **Would you like to receive newsletters from the SOPHIE project?** (if so, please provide you e-mail address) |  |
