# Supplementary material for: Innovative actions in oceans and human health for Europe
Source: Health Promot Int. 2021 Dec 22;38(4):daab203. doi: 10.1093/heapro/daab203 (PMC10405041; doi:10.1093/heapro/daab203)
Supplement: daab203_Supplementary_Data [file daab203_supplementary_data.zip › InnovativeActionsOceansHealth_Appendix3new.docx]

**Title**

Innovative actions in Oceans and Human Health for Europe

# SUPPLEMENTARY APPENDIX 3

Interview guide for key informants in Europe

| **Introductory questions** | *If initiator*: What motivated you to start up this initiative?   - Was there an issue unaddressed that you aimed to tackle?   *If participant*: What was the motivation for this initiative to take action?   - Was there an issue that was unaddressed that it meant to tackle? |
| --- | --- |
|  | What are the main positive impacts of the initiative/innovation on oceans/human health?   - What do you think were key success factors? |
| **Insight in other innovations** | What other OHH innovations do you know of that are present in your region/country? |
|  | Do you know of any similar innovations to yours in other countries? |
|  | Do these innovations have a website or social media channel? If not, can you share a contact? |
| **Missing topics** | Can you think of any examples of (environmental/health) issues that are not yet covered by innovations?   - What could be an innovative solution to that issue according to you? |
| **Link back to innovations** | What do you think about upscaling local OHH innovations throughout the country/region/continent?   - How transferrable do you think the innovations are? - How effective do you think they are? |
|  | Is there a platform for local innovations in your country/region that can be contacted for further information? |
| **Closing question** | Do you have any questions or is there anything you would like to add? |
