# Supplementary material for: Innovative actions in oceans and human health for Europe
Source: Health Promot Int. 2021 Dec 22;38(4):daab203. doi: 10.1093/heapro/daab203 (PMC10405041; doi:10.1093/heapro/daab203)
Supplement: daab203_Supplementary_Data [file daab203_supplementary_data.zip › InnovativeActionsOceansHealth_Appendix4new.docx]

**Title**

Innovative actions in Oceans and Human Health for Europe

# SUPPLEMENTARY APPENDIX 4

List of 150 innovative actions, listed by their response to the various categories of the *Drivers, Pressures, State, Impact* and *Responses* framework (DPSIR). The innovators of the shaded actions have been interviewed.

|  | **Name** | **Website** |
| --- | --- | --- |
| ***Response* to DPSIR-category: D*river*** | | |
| D | Co-development of Climate Services for Adaptation to Changing Marine Ecosystems (CoCliME) | <https://www.coclime.eu/> |
| D | Stichting Anemoon | <https://www.anemoon.org/> |
| D | Marine CoLABoration | <https://marinecolab.org/> |
| D | Air Powered Sampling for Purse Seine Fisheries | <https://motherboard.vice.com/en_us/article/z4m8yx/the-net-that-lets-fishermen-preview-their-catch> |
| D | Valuing the health benefits of physical activities in the marine environment | <https://www.sciencedirect.com/science/article/pii/S0308597X15002936> |
| D | Ocean Health Index | <http://ohi-science.org/> |
| D | Chiringuitos Responsables / Responsible Beach Bars | <http://www.chiringuitosyvoluntarios.es/chiringuito-responsable/> |
| D | Blue Flag | <http://www.blueflag.global/> |
| D | Save the Ocean Feed the World | <http://oceana.org/our-campaigns/save_oceans_feed_world/campaign> |
| D | AKTEA | <http://akteaplatform.eu/> |
| D | SAFE SEA | <https://estlat.eu/en/estlat-results/safe-sea.html> |
| D | ESTLAT Harbours | <https://estlat.eu/en/estlat-results/estlat-harbours.html> |
| D | EDMAKTUB | <http://www.edmaktub.org/en/2016/06/drones-for-cetacean-research/> |
| D | Sofar Ocean | <https://www.sofarocean.com/> |
| ***Response* to DPSIR-category: *environmental Pressure*** | | |
| EP | PRIMROSE | <https://www.atlanticarea.eu/project/12> |
| EP | Quut | <https://www.allesvanquut.nl/> |
| EP | International Convention for the Prevention of Pollution from Ships (MARPOL) | <http://www.imo.org/en/About/Conventions/ListOfConventions/Pages/International-Convention-for-the-Prevention-of-Pollution-from-Ships-(MARPOL).aspx> |
| EP | Maritime Blockchain Labs | <https://www.maritimeblockchainlabs.com/> |
| EP | By the ocean we unite | <https://www.bytheoceanweunite.org/> |
| ***Response* to DPSIR-category: *human Pressure*** | | |
| HP | The Plastic Soup Foundation | <https://www.plasticsoupfoundation.org/> |
| HP | Loliware | <https://www.loliware.com/> |
| HP | Ocean Witness | <https://oceanwitness.org/> |
| HP | SeaWalls - Artists for oceans | <https://www.pangeaseed.foundation/sea-walls/> |
| HP | One Ocean FM | <http://www.oneocean.fm/> |
| HP | Smartfin surfing for citizen science | <https://smartfin.org/> |
| HP | Capturing Our Coast | <https://capturingourcoast.co.uk/> |
| HP | Ocean Campus - Surfrider Foundation | <http://en.oceancampus.eu/> |
| HP | Good Fish Guide | <https://www.mcsuk.org/responsible-seafood/> |
| HP | Blue School programme | <http://escolaazul.pt/en> |
| HP | EDUCO2CEAN project | <http://www.educo2cean.org/> |
| HP | Youth Making Ripples - film competition for youngsters | <http://youthmakingripples.org/> |
| HP | Coastwatch Campaign | <http://coastwatch.org/europe/survey/> |
| HP | World Ocean Day | <http://www.worldoceansday.org/> |
| HP | Sea Change awareness campaign "Our Ocean, Our Health" | <http://www.seachangeproject.eu/our-ocean-our-health> |
| HP | The European Marine Science Educators Association (EMSEA) | <http://www.emsea.eu/default.php> |
| HP | Pesce Ritrovato / Rediscovered Fish | <http://www.pesceritrovato.it/> |
| HP | Fish Forward Project | <https://www.fishforward.eu/en/fish-forward-description/> |
| HP | BioWatch | <http://www.bio-watch.com/> |
| HP | Hebridean Whale and Dolphin Trust | <https://hwdt.org/> |
| HP | Sea First | <https://www.seafirst.nl/> |
| HP | ResponSEAble | <https://www.responseable.eu/> |
| HP | Watertrek | <https://www.watertrek.org/home/> |
| ***Response* to DPSIR-category: *environmental State*** | | |
| ES | CERES | <https://ceresproject.eu/> |
| ES | Project Baseline | <http://projectbaseline.org/> |
| ES | Exxpedition | <http://exxpedition.com/> |
| ES | Adopt a float | <http://www.monoceanetmoi.com/web/index.php/en/adopt-a-float-home> |
| ES | Reef Design Lab | <http://www.reefdesignlab.com/> |
| ES | CSI - Community Seagrass Initiative | <http://www.csi-seagrass.co.uk/> |
| ES | Save the waves | <https://www.savethewaves.org/> |
| ES | LEMA tool | [www.lifelema.eu](http://www.lifelema.eu) |
| ES | Seabin | <https://www.seabinproject.com/> |
| ES | ORCA | <https://www.orcaweb.org.uk/> |
| ES | SeaSearch | <http://www.seasearch.org.uk/index.html> |
| ES | Surfers against Sewage | <https://www.sas.org.uk/> |
| ES | Ghost fishing | <https://www.ghostfishing.org/recycling/> |
| ES | Recycled Park | <http://recycledpark.com/> |
| ES | Life Posidonia Andalucia project - Conservation of Posidonia oceanica | <http://ec.europa.eu/environment/life/project/Projects/index.cfm?fuseaction=search.dspPage&n_proj_id=3829> |
| ES | Kosterhavet Marine Protected Area | <https://www.vastsverige.com/en/kosterhavet/>  <http://extra.lansstyrelsen.se/kosterhavet/SiteCollectionDocuments/sv/english/kosterhavet-national-park.pdf> |
| ES | Healthy Seas initiative | <http://healthyseas.org/> |
| ES | Levävahti - algae watch | <https://environmentalsystemsresearch.springeropen.com/articles/10.1186/s40068-014-0024-8> |
| ES | Dive the North Sea Clean - removal of ghost fishing nets from wrecks | [www.duikdenoordzeeschoon.nl](http://www.duikdenoordzeeschoon.nl) |
| ES | Plogga | Facebook and Instagram @Plogga, <https://ploggase.firebaseapp.com/for-foretag/>, <https://spark.adobe.com/page/i4iKpksF47Qkm/> |
| ES | Citizens' Network for the Observation of Marine Biodiversity - COMBER (Greece) | <https://comber.hcmr.gr> |
| ES | Volvo Ocean Race - Sustainability Programme | <https://www.volvooceanrace.com/en/sustainability.html> |
| ES | Environmental kayak (miljoekajakken) | <https://kulturhavn365.dk/miljoekajakken/> |
| ES | International Coastal Cleanup | <https://oceanconservancy.org/trash-free-seas/international-coastal-cleanup/> |
| ES | Dive Against Debris | <http://www.projectaware.org/diveagainstdebris> |
| ES | MARLISCO - Raising awareness and co-responsibility for marine litter in Europe | [www.marlisco.eu](http://www.marlisco.eu) |
| ES | Toxicity profiling of marine surface sediments: A case study using rapid screening bioassays of exhaustive total extracts, elutriates and passive sampler extracts | |
| ES | Marine Litter Watch | <https://www.eea.europa.eu/themes/water/europes-seas-and-coasts/marine-litterwatch> |
| ES | Fishing for Litter - Engaging fishermen in the removal of litter from the sea | <http://www.fishingforlitter.org.uk/what-is-fishing-for-litter> |
| ES | The New Raw | <https://thenewraw.org/> |
| ES | PlanBlue | <https://www.planblue.com/> |
| ES | Secchi Disk | <http://www.secchidisk.org/> |
| ES | Gökçeada Marine Underwater Park | <http://gokceadasualtiparki.org/index.php?lang=en> |
| ES | REEFS (Research and REstoration of the Essential Filters of the Sea) | <http://reefsproject.net/> |
| ES | Black Sea Watch Project | <http://blackseawatch.org/Default.aspx> |
| ES | Crab Watch | <http://www.seachangeproject.eu/seachange-about-4/crab-watch> |
| ES | ECOncrete | <https://econcretetech.com/> |
| ES | The Marine Foundation | <http://themarinefoundation.org/> |
| ES | The Shore Thing | <https://www.mba.ac.uk/shore_thing/index.htm> |
| ES | Ecoduikers | <https://www.ecoduikers.be/?fbclid=IwAR1V4CuZImOgcz0Q-u_uWnmPinBi3Dv0k_AHCTWoy0weBELucMp1JbgHxl0> |
| ES | Sea Ranger Service | <http://www.searangers.org/en/> |
| ES | Protect Blue | [www.protect.blue](http://www.protect.blue) |
| ES | The Great Bubble Barrier | <https://thegreatbubblebarrier.com/en/> |
| ES | OCEARCH | <https://www.ocearch.org/> |
| ES | REEF Check | <https://www.reefcheckmed.org/english/underwater-monitoring-protocol/webgis-map/> |
| ***Response* to DPSIR-category: *human State*** | | |
| HS | Seafarers Hospital Society | <https://seahospital.org.uk/> |
| HS | The mission to seafarers | <https://www.missiontoseafarers.org/> |
| HS | Swim Guide | <https://www.theswimguide.org/> |
| HS | The Jellyfish App | <https://thejellyfishapp.com/> |
| HS | Safe Haven Ireland | <https://www.safehavenireland.com/> |
| HS | Sea Sanctuary | <https://seasanctuary.org.uk/> |
| HS | The Blue New Deal | <http://www.bluenewdeal.org/about/> |
| HS | Low Impact Fishermen of Europe (LIFE) | <http://lifeplatform.eu/> |
| HS | Surfing Medicine International | <http://www.surfingmed.com/> |
| HS | Welcome Wave | <https://www.joe.ie/life-style/welcome-wave-636783> |
| HS | Safe Water Sports | <https://safewatersports.com/en/> |
| HS | KOSTASystem, | <http://www.kostasystem.com/> |
| HS | Mobile qPCR *E.coli* |  |
| HS | Expeditie Juttersgeluk / "Beachcombers delights" | <http://www.juttersgeluk.nl/expeditie/> |
| HS | ECsafeSEAFOOD Project | <http://www.ecsafeseafood.eu/> |
| HS | Med Jelly-Risk / Spot the jelly Fish | <http://jellyrisk.eu/en/#.Wuh95KSFOpo>  <http://www.ioikids.net/jellyfish> |
| HS | Bathing Water Quality Forecasting System | <http://environment.data.gov.uk/bwq/profiles/> |
| HS | Amphicruiser Rescue | <https://www.amphicruiser.com/rescue/> |
| HS | Autism on the water | <https://www.facebook.com/pg/AutismOnTheWater/about/?ref=page_internal> |
| HS | Obonjan Island | <https://www.obonjan-island.com/> |
| HS | Sirens | <http://www.inspiresirens.org/> |
| HS | Deptherapy | <http://deptherapy.co.uk/index.html> |
| HS | SUPYogaPilates | <http://supyogapilates.com/> |
| HS | Research Project Sea-Kayaking & Wellbeing, Dissertation 2019 | n/a at this stage |
| HS | Sailing into Wellness | <https://www.sailingintowellness.ie/> |
| HS | Wave by wave | <http://wavebywave.org/index.html> |
| HS | SurfABLE Scotland | <https://friendlyaccess.org/what-we-do/surfable-scotland/> |
| HS | Liquid therapy | <http://liquidtherapy.ie/> |
| HS | Healing Waves | <http://www.healingwaves.org.je/> |
| HS | WetWheels | <https://www.wetwheels.je/> |
| HS | Oceans of Hope | <http://www.oceansofhope.org/> |
| HS | Ocean Recovery | <https://oceanrecoverycentre.com/> |
| HS | Adria Experience | <https://adriaexperience.com/> |
| HS | Coral Biome | <https://www.coralbiome.com/pharma/> |
| ***Response* to DPSIR-category: *Impact*** | | |
| I | Scott Haldane Foundation | <https://www.scotthaldane.nl/nl/> |
| I | Mater Museoa | <http://www.matermuseoa.com/> |
| I | CoolRoute | <http://www.sailcoolroute.eu/> |
| I | Costa Balenae | <http://www.costabalenae.it/en/home> |
| I | EcoMarine Malta | <http://www.ecomarinemalta.com.mt/> |
| I | Edulis | <http://www.aqua.ugent.be/edulis> |
| I | The Outdoor Swimming Society | <https://www.outdoorswimmingsociety.com/> |
| I | School at Sea | <https://www.schoolatsea.com/> |
| I | Wild Swimming | <http://www.wildswimming.co.uk/> |
| I | SUPKids | <https://sup-kids.com/> |
| I | Surf Therapy | <https://intlsurftherapy.org/> |
| I | Vies Braves | https://viesbraves.com/en/ |
| I | Seafarm project - Seaweed farming in Sweden | <https://www.kth.se/en/forskning/artiklar/alger-framtidens-allroundmat-1.612780>  <http://www.seafarm.se/web/page.aspx?refid=135> |
| I | The Wave Project - surf therapy for disabled and vulnerable young people | [www.waveproject.co.uk](http://www.waveproject.co.uk) |
| I | Community "Oceans and Human Health Chair"/"Càtedra Oceans i Salut Humana" | <http://www.oceanshealth.udg.edu/ca/qui-som.html> |
| I | My Ocean Sampling Day | https://www.microb3.eu/osd.html, <http://www.assembleplus.eu/research/ocean-sampling-day-2018> |
| I | Mr. Goodfish Campaign | <https://www.mrgoodfish.com/en/> |
| I | Stichting Noordzeeboerderij | <https://www.noordzeeboerderij.nl/> |
| I | PharmaSea | <http://www.pharma-sea.eu/> |
| I | High Quality Whale Watching Certificate | <http://www.whale-watching-label.com/accueil> |
| I | Yayakarsa | <http://www.yayakarsa.org/index.php/en-US/> |
| I | Slow Mill | <http://www.slowmill.nl/> |
| I | Waddenwier | <https://www.waddenwier.com/> |
| I | Seaweed for Food and Feed | <https://www.noordzeeboerderij.nl/projecten> |
| I | Swansea Bay Tidal Lagoon | <https://www.marineenergywales.co.uk/marine-energy-in-wales/projects/tidal-lagoon-swansea-bay/> |
| I | VoyageVert | [www.voyagevert.org/](http://www.voyagevert.org/) |
| I | WaveSub | <http://marinepowersystems.co.uk/> |
| I | Coastal hiking | <https://estlat.eu/en/estlat-results/coastal-hiking.html> |
| I | CITiZAN | <https://www.citizan.org.uk/> |
