# Supplementary material for: Innovative actions in oceans and human health for Europe
Source: Health Promot Int. 2021 Dec 22;38(4):daab203. doi: 10.1093/heapro/daab203 (PMC10405041; doi:10.1093/heapro/daab203)
Supplement: daab203_Supplementary_Data [file daab203_supplementary_data.zip › InnovativeActionsOceansHealth_Appendix5new.docx]

**Title**

Innovative actions in Oceans and Human Health for Europe

# SUPPLEMENTARY APPENDIX 5

Innovative actions as *Responses* to environmental issues

Innovative actions in each of the categories of the DPSIR framework, addressed various environmental issues. Reviewing how the various types of DPSI *Responses* were divided over the different environmental issues showed how and why the actions targeting a certain type of environmental issues addressed some categories of DPSI more (or less) than others. In Figure S3 the baseline in grey shows the distribution of innovative actions over each of the DPSI categories. Values above the baseline indicate that a certain environmental issue was disproportionally targeted by a response to that particular driver. This is best explained for the most targeted issues: ‘loss of biodiversity’, ‘plastic pollution’, ‘commercial fish stock depletion’ and ‘climate change’.

[insert here - Figure S5. Environmental issues addressed in each of the DPSIR categories of 150 innovative actions on oceans and human health in Europe]

Hence, the innovative actions to reduce the ‘loss of biodiversity’ (pink dashed line in Figure S5, targeted by 33% of the actions) show higher peaks at the environmental *State* and *Drivers*, as compared to the baseline. This makes sense as these were efforts to restore, conserve and protect marine ecosystems and their biodiversity, i.e. targeting the environmental *State*, while at the same time requiring policy support, typically targeting the *Drivers*.

The plain orange line for innovative actions on ‘plastic pollution’ (targeted by 31% of actions) clearly peaks at environmental *Pressures*. This means that the issue of plastic pollution was mainly addressed in actions that prevented the environmental *Pressure* of plastic waste disposal into the (marine) environment. The actions on ‘plastic pollution’ also responded disproportionally to *Drivers*, through policy. Other innovative actions removed and reduced plastic waste to prevent it from doing harm to the human health *State*. While many actions aimed at removing plastic pollution from the ocean to improve the environmental *State*, this was below the average, and much less than actions that targeted the loss of biodiversity.

For ‘commercial fish stock depletion’ (plain pink line in Figure S5, targeted by 22%), the actions addressed *Impact*, *Drivers* and human *State* more than the baseline. This was done by, respectively, restoring ecosystem services, by supporting regulations and policy making, and by offering sustainably caught fish or alternative healthy seafoods.

Actions that addressed ‘climate change’ (19% of the actions, the purple plain line in Figure S5) responded more than others (though less so than those aimed at plastic pollution) to environmental *Pressures* through prevention of atmospheric emissions and extractive practices by innovating in renewable marine energy. Additionally, they responded to *Impact* by enhancing ecosystem services such as climate regulation and sustainable, renewable energy.

# FIGURE CAPTION

Figure S5. Environmental issues addressed in each of the DPSIR categories of 150 innovative actions on oceans and human health in Europe.
